# The role of testosterone, the androgen receptor, and hypothalamic-pituitary–gonadal axis in depression in ageing Men

**DOI:** 10.1007/s11154-022-09767-0

**Published:** 2022-11-22

**Authors:** Richard L. Hauger, Ursula G. Saelzler, Meghana S. Pagadala, Matthew S. Panizzon

**Affiliations:** 1 Center of Excellence for Stress and Mental Health (CESAMH), VA San Diego Healthcare System, San Diego, CA USA; 2 Center for Behavior Genetics of Aging, Department of Psychiatry, School of Medicine, University of California San Diego, La Jolla, CA USA; 3 Medical Scientist Training Program, School of Medicine, University of California San Diego, La Jolla, CA USA; 4 Biomedical Science Program, School of Medicine, University of California San Diego, La Jolla, CA USA

**Keywords:** Testosterone, Androgen receptor, Hypogonadism, Androgen deprivation therapy, Testosterone replacement therapy, Depression, Major depressive disorder

## Abstract

Considerable research has shown that testosterone regulates many physiological systems, modulates clinical disorders, and contributes to health outcome. However, studies on the interaction of testosterone levels with depression and the antidepressant effect of testosterone replacement therapy in hypogonadal men with depression have been inconclusive. Current findings indicate that low circulating levels of total testosterone meeting stringent clinical criteria for hypogonadism and testosterone deficiency induced by androgen deprivation therapy are associated with increased risk for depression and current depressive symptoms. The benefits of testosterone replacement therapy in men with major depressive disorder and low testosterone levels in the clinically defined hypogonadal range remain uncertain and require further investigation. Important considerations going forward are that major depressive disorder is a heterogeneous phenotype with depressed individuals differing in inherited polygenic determinants, onset and clinical course, symptom complexes, and comorbidities that contribute to potential multifactorial differences in pathophysiology. Furthermore, polygenic mechanisms are likely to be critical to the biological heterogeneity that influences testosterone-depression interactions. A genetically informed precision medicine approach using genes regulating testosterone levels and androgen receptor sensitivity will likely be essential in gaining critical insight into the role of testosterone in depression.

## Introduction

**Fig. 1:**
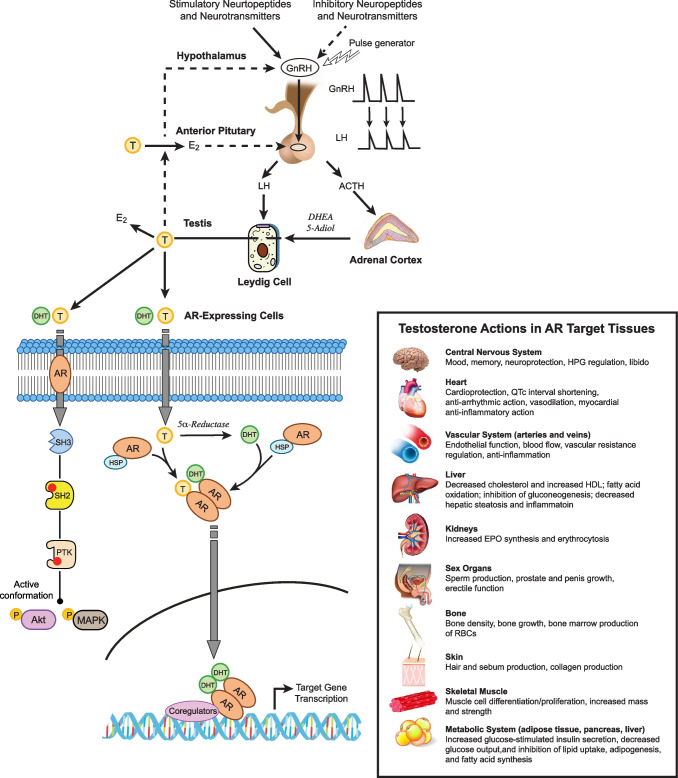
Regulation of the hypothalamic-pituitary–gonadal axis, testicular synthesis of androgens, and physiological actions of testosterone resulting from androgen receptor signaling in targeted tissues. The complex, multilevel regulation of the hypothalamic-pituitary–gonadal axis is mediated by stimulatory and inhibitory neurocircuits acting on gonadotropin-releasing hormone (GnRH) neurons in the arcuate/infundibular nucleus and medial preoptic area of the hypothalamus. Testosterone secreted by the testis exerts negative feedback control of hypothalamic GnRH release, while estradiol formed by 5α-reductase conversion of testosterone exerts negative feedback control of anterior pituitary luteinizing hormone (LH) secretion. Synthesis of testosterone and dihydrotestosterone (DHT) by the testis is stimulated by LH activating G protein-coupled LH receptors in Leydig cells. ACTH-stimulated synthesis of DHEA, 5-Adiol, and androstenedione by adrenocortical cells may contribute to testicular synthesis of testosterone and DHT via the “backdoor” pathway, although some studies indicate that DHEA and 5-Adiol secreted by the adrenal cortex may serve as substrates for peripheral conversion of testosterone by androgen receptor-regulated target tissues. Testosterone and DHT secreted by the testis bind to and activate the androgen receptor (AR) expressed in peripheral organs and the central nervous system. The slower genomic actions resulting from classical, canonical androgen receptor signaling involve dissociation of cytosolic AR from heat shock proteins, translocation of AR with chaperones to the nucleus, and then binding of AR and co-regulators to androgen response elements on target genes to activate or repress their expression. In contrast, rapid, non-genomic actions result for membrane androgen receptors signaling via downstream Akt and ERK-MAP kinase pathways. The complex mechanisms governing testosterone hormone action regulate many physiological systems, modulate clinical disorders, and contribute to health outcome. The dotted line indicates an inhibitory action, while the solid line indicates a stimulatory action

Testicular androgens have crucial roles in physiological homeostasis, health outcome, and disease pathophysiology. Testosterone and the more biological active androgen, dihydrotestosterone (DHT), formed by conversion of testosterone by 5α-reductase, act as the primary sex hormones in men regulating male sexual development during puberty and spermatogenesis and sexual function in adulthood [1–3] (Fig. 1). Other classical, well-established roles of testosterone include stimulation of erythropoiesis and maintenance of muscular strength and volumetric bone density mass [4, 5] (Fig. 1). Subsequent research, however, has discovered that androgens have more extensive physiological actions regulating cardiovascular, metabolic, hepatic, and immune systems and, importantly, the central nervous system [6–10] (Fig. 1).

The prevalence of major depressive disorder is two-folder higher in women compared to men suggesting that physiological levels of testosterone in the healthy range may reduce the risk of depression [11]. Preclinical research has provided further evidence that androgens may reduce the risk of depression in men due to their antidepressant and neuroprotective actions in the hippocampus, limbic system, and other brain regions regulating mood [12, 13]. Considerable work has shown that low testosterone levels, clinical hypogonadism, pharmacologically induced testosterone deficiency by androgen deprivation therapy, and androgen receptor antagonist treatment are significantly associated with depression in men, although some studies have not observed this effect. An important research question is whether low testosterone levels are a trait biomarker for the depression risk, or a state biomarker associated with a major depressive episode and its severity. Alternatively, however, low testosterone levels may be a result of co-morbid medical conditions associated with depression. The focus of this review will assess the role of testosterone in mood regulation regarding the above important issues.

## Testosterone levels, hypogonadism, and depression

### Testosterone decline and hypogonadism during aging

In young, healthy men, circulating levels of total testosterone range from 300–1000 ng/dl (10.4–34.7 nmol/L SI units) with 0.5% to 3.0% being free testosterone unbound to sex hormone binding globulin (SHBG) or albumin [1, 2]. The Baltimore Longitudinal Study of Aging has reported that 80% of 60-year-old men and 50% of 80-year-old men exhibit total testosterone levels within the normal range of young men [14, 15]. Other men, however, experience a substantial age-related decline in total testosterone into the clinical hypogonadal range below 280–300 ng/dl (9.7–10.4 nmol/L SI units). Many early cross-sectional studies reported that total testosterone levels in men begin to decline at the age of 40 by a rate of 0.4% per year [15, 16]. Other cross-sectional research found that free testosterone levels decreased more rapidly at a rate of 1.5–2.0% in older men due to the age-dependent upregulation of SHBG [16]. A smaller number of longitudinal studies reported a greater rate of testosterone decline during aging with total testosterone decreasing by 1–2% per year [15, 16].

Although most studies on testosterone decline during aging have involved older men, a recent longitudinal study of young, healthy men (average age 34) found that the age at baseline did not predict changes in the trajectories of testosterone, dihydrotestosterone, androstenedione, and estradiol measured by LC–MS/MS mass spectrometry over a twelve-year period [17]. Furthermore, gonadotropin secretion was upregulated and the testosterone/ luteinizing hormone ratio was decreased indicating declining Leydig cell function despite these men being young. BMI was negatively associated with circulating levels of total and free testosterone, DHT, androstenedione, and estradiol [17].

Research has shown that the age-related decrease in testosterone is mediated by several important mechanisms: (1) impaired luteinizing hormone (LH) receptor signaling via the protein kinase A-cyclic AMP pathway; (2) dysregulation of cholesterol transport and metabolism in mitochondrial due to oxidative stress [18], (3) the attrition of Leydig cells [128]. Furthermore, it is well established that the rate of testosterone decline can be accelerated by modifiable lifestyle factors including obesity and alcohol consumption. Several studies have shown that certain chronic medical disorders, especially type 2 diabetes, may be more important in promoting testosterone decline than increasing age [2, 16, 19, 20]. Importantly, recent research has shown that genetic factors can regulate the trajectory of testosterone during aging [21–23].

### Relationship of circulating levels of testosterone and depression

Early studies discovered a significant association  of increasing severity of major depressive disorder with low circulating levels of total testosterone in men [24]. Subsequently, observational, cross-sectional, or longitudinal studies reported an inverse relationship of depression scores in men with circulating testosterone levels in the low physiological and hypogonadal ranges, while other studies did not find a relationship of depressive symptoms and testosterone levels [20, 25, 26]. In 1999, the Rancho Bernardo Study reported that lower plasma levels of bioavailable testosterone, calculated using SHBG and albumin, and dihydrotestosterone were associated with higher Beck Depression Inventory (BDI) scores in their large cohort of community-dwelling older men (50 to 89 years) [27]. Total testosterone and estradiol were not significantly associated with depressive symptoms. It is also important to note that none of the men in the Rancho Bernardo Study had testosterone levels in the hypogonadal range. Higher estradiol levels have been reported to be associated with depression in young, obese men. Further investigation is required to elucidate the role of estradiol and its interaction with testosterone in depression especially in older men with hypogonadal testosterone level, which has been difficult to study due in part to mass spectrometry being necessary for specific, sensitive, and quantitative measurement. In addition, the roles of dihydrotestosterone, androstenedione, and other androgenic steroids in depression also warrants further investigation.

### Relationship of testosterone deficiency in hypogonadism and depression

Many clinical symptoms of hypogonadism resemble the symptoms of major depressive disorder. Hypogonadal men frequently experience a depressed mood, anhedonia, fatigue, and cognitive impairment, which are four of the five diagnostic criteria A specified for major depressive disorder in the Fifth Edition of the Diagnostic and Statistical Manual of Mental Disorders [25]. In 2018, the Endocrine Society Clinical Practice Guideline established criteria for hypogonadism requiring that two morning serum testosterone levels are below 280–300 ng/dl (9.7–10.4 nmol/L SI units) [1]. When clinical criteria for hypogonadism are used, consistent  increases in depressive symptomatology and incidence of clinical depression  have been reported in hypogonadal men with confirmed testosterone deficiency compared to eugonadal men with testosterone levels in the normal physiological range. In 2004, a careful study using the Department of Veterans Affairs Healthcare System electronic medical record and a Kaplan–Meier survival analysis reported a two-year incidence of major depressive disorder of 21.7% and a shorter time to the development of depression [OR = 3.5, p = 0.01] in men with an average age of 64.5 years and a stringent diagnosis of hypogonadism defined as total testosterone below 200 ng/dl (6.93 nmol/L SI units) compared to eugonadal men [28]. In 2005, using less stringent cutoff for hypogonadal levels defined as total testosterone below 250 ng/dl (8.67 nmol/L SI units), older, hypogonadal men (average age 69 years) with no history of depression had a higher incidence of a depressive episode (by ICD-9 diagnosis) and a more rapid onset of depression [adjusted HR = 2.1, p = 0.002] over a two-year period compared to eugonadal men [28]. Increasing age and a higher number of co-morbid medical disorders were important factors [28].

In 2006, a Canadian study reported that total and bioavailable testosterone were significantly lower in middle-aged depressed men (40–65 years) who had considerably higher BDI and Hamilton depression scores than men enrolled in the Rancho Bernardo Study [29]. Furthermore, using a logistic regression, this study found that high depression scores were present in 61% of men with hypogonadism compared to only 14% of eugonadal men [29]. The cross-sectional Health in Men Study (HIMS) in Australia reported that the risk of depression increased threefold in men with free testosterone level below 60 pg/ml compared to men with a free testosterone level above 100 pg/ml [30]. These findings emphasize that the degree of testosterone deficiency is important. Likewise, in an adjusted linear regression analysis, the prospective Longitudinal Aging Study Amsterdam observed greater depressive symptoms in men with the lowest quartile of calculated free testosterone compared to men in the highest free testosterone quartile [31]. Furthermore, there was  twofold increase in the development of depression [HR = 1.989] in men with free testosterone in the hypogonadal range (< 220 pmol/L SI units; < 63.4 pg/ml) over a ten-year follow-up period [31].

In 2016, the Health in Men Study  provided further support for the association of hypogonadism and depression by finding that total testosterone levels below 6.41 nmol/L (185 ng/dl) predicted a high risk of developing incident depression in older men (71–88 years) over a ten-year period after adjusting for age, cardiovascular disorders, and diabetes [HR = 1.86] [32]. Men with normal total testosterone levels had a considerably longer depression-free survival period [32]. This study also reported that low levels of dihydrotestosterone, estradiol, and free testosterone (calculated) did not confer risk for developing incident depression. In addition to being a prospective study, another strength of the HIMS study was measuring total testosterone levels using LC–MS/MS mass spectrometry, which is a critical methodology for accurately measuring hypogonadal testosterone levels [32]. Importantly, a recent investigation of 169,886 male participants (40–69 years) without a history of depression in the prospective UK Biobank study also found men with hypogonadal total testosterone levels (< 6 nmol/L) had a higher five-year incidence of major depressive episode [adjusted OR = 1.60] [33]. The association of major depressive disorder incidence with testosterone levels in the hypogonadal range had the largest effect size among the 57 laboratory tests analyzed in the UK Biobank.

### Hypogonadotropic hypogonadism and depression

A previous study found that young men with congenital hypogonadotropic hypogonadism due to a GnRH deficiency who had very low testosterone levels (78 ng/dl; 2.70 nmol/L SI units) compared to normal controls (483 ng/dl; 16.74 mmol/L SI units) exhibited a high incidence of depression [34]. When hypogonadotropic hypogonadal men were treated with testosterone replacement therapy, their Beck depression score decreased by 90% and was similar to normal male controls [34].

### Meta-analyses of testosterone levels and depression

An earlier meta-analysis of five studies found a significant association of total testosterone levels in the hypogonadal range with Hamilton depression (HAM-D) scores [Z = -3.84; p = 0.0001] [35]. A recent meta-analysis of seven studies involving 1,452 men with mean ages ranging from 36 to 74 years demonstrated that low testosterone levels were significantly associated with major depressive disorder [Z = -2.53; p = 0.012] [36]. These meta-analyses further strengthen the concept that clinical hypogonadism confers a high risk for depression in men.

## Hypothalamic-pituitary–gonadal axis in depression

### Regulation of the hypothalamic-pituitary–gonadal axis

Dysregulation of the hypothalamic-pituitary–gonadal (HPG) axis has been observed in patients with major depressive episodes. Androgen regulation of the hypothalamic-pituitary–gonadal (HPG) axis is critical for homeostatic regulation of synthesis and secretion of testosterone and the most potent androgen dihydrotestosterone (DHT) by the testis (Fig. 1). Because circulating levels of gonadotropins do not change when pituitary androgen receptors are knocked out in transgenic mice, gonadotrophs in the anterior pituitary do not appear to be a site for testosterone negative feedback [37]. Increasing testosterone levels have been found to inhibit hypothalamic GnRH release via classical negative feedback thereby reducing anterior pituitary secretion of LH and FSH and their stimulation of testosterone steroidogenesis [38]. After research indicated GnRH neurons do not express androgen receptors, kisspeptin and its G protein-coupled receptor KISS1R were discovered as important regulators of GnRH neurons [39]. Testosterone feedback without interacting directly with GnRH neurons targets AR-expressing kisspeptin neurons in the arcuate nucleus of the hypothalamus to negatively regulate pulsatile GnRH release and the HPG axis [38, 40].

In addition to regulating the HPG axis via kisspeptin signaling, testosterone also regulates kisspeptin neurons in the amygdala and hippocampus [39]. Interestingly, kisspeptin has been found to have an antidepressant action possibly by modulating brain serotonergic neurons [41]. Considering that the brain serotonergic neuronal system has a critical role in depression and antidepressant treatment, the interaction of testosterone and kisspeptin neurotransmission may have an unrecognized role in major depressive disorder. Other research has shown that testosterone may exert an antidepressant action by activating androgen receptor MAPK-ERK2 signaling in the hippocampus [12].

### Dysregulation of the hypothalamic-pituitary–gonadal axis and depression

In a circadian study, daytime and nocturnal total testosterone levels and the 24-h mean testosterone secretion were significantly lower in men with severe major depressive episodes based on high Hamilton scores and high 24-h mean cortisol secretion [42]. The role of hypothalamic–pituitary–adrenal hypersecretion observed in severe major depressive episodes and the well-known ability of high cortisol to suppress the hypothalamic-pituitary–gonadal axis in the relationship of testosterone and depression requires further investigation.

Subsequent neuroendocrine research including meta-analyses  have found that basal testosterone levels and 24-h testosterone secretion are abnormally low in men with major depressive episodes [25, 36, 43]. Basal secretion of LH and FSH, LH pulse frequency, and GnRH-stimulation gonadotropin secretion by the anterior pituitary are not altered in major depressive disorder indicating that anterior pituitary gonadotropin dysregulation may not contribute to low testosterone levels [36, 43, 44]. A recent meta-analysis of hypothalamic-pituitary–gonadal dysregulation in depression raised the caveat that new LH and FSH assays with greater sensitivity and improved quality control should be used to reassess the role of gonadotropin secretion  in depression.

## Androgen deprivation therapy and depression

### Androgen deprivation therapy and testosterone levels

Androgen deprivation therapy (ADT) is the first line treatment for advanced, metastatic, and recurrent prostate cancer due to its ability to dramatically reduce circulating testosterone. ADT involves treatment with a gonadotrophin-releasing hormone (GnRH) superagonist to desensitize and downregulate pituitary GnRH receptors, thereby depleting testosterone [45, 46]. The result is a profound reduction in circulating levels of testosterone and dihydrotestosterone, by up to 97% without any change in SHBG. Importantly, ADT produces a more severe testosterone deficiency decreasing circulating testosterone to castration levels below 20 ng/dl (0.69 nmol/L SI units), in contrast to the considerably smaller reduction in testosterone levels defining clinical hypogonadism (< 280–300 ng/dl; < 9.7–10.4 nmol/L SI units) [45, 46]. ADT decreases to a lesser extent (~ 40%) the secretion of adrenocortical androgens DHEA, its sulfate metabolite DHEA-S, and androstenedione, which is regulated by ACTH [45] (Fig. 1). ADT results in many adverse physiological effects, far more frequent and intense than occurring in clinical hypogonadism, which includes severe fatigue, increased adiposity and obesity, dyslipidemia, insulin resistance, cardiovascular dysregulation, sarcopenia, osteoporosis and fractures, sexual dysfunction, and increased inflammation [47, 48]. These systemic changes can lead to coronary artery disease, type 2 diabetes, and dyslipidemia, and increase the risk of developing depression [49–51].

### Studies of androgen deprivation therapy and depression

The rate of depression is significantly higher in men with prostate cancer compared to cancer-free men [52]. Treatment of prostate cancer with radical prostatectomy or radiation therapy has also been associated with depression [52, 53]. However, androgen deprivation therapy has been shown to have a substantially stronger  induction of depression. The association of androgen deprivation therapy and depression represents the most extensively studied psychiatric outcome variable due to its detrimental impact on survivorship [49, 51]. Androgen deprivation therapy has been reported to provoke depressive symptoms and increase the incidence of major depressive episodes in many but not all studies. Since 2000, several small, cross-sectional studies have reported that ADT treatment for 3 to 12 months is associated with significant increases in self-reported depression compared to men with prostate cancer without ADT or healthy controls [50, 54–61]. One early study reported the prevalence of major depressive disorder based on DSM-4 criteria in older men (> 65 years) treated with ADT was 12.8%, which was eightfold higher than the national prevalence rate in men at the same age not receiving ADT [59]. In an Asian cohort, the rate of incident depression over a three-year period was 13.9% in men with prostate cancer treated with ADT who had no prior diagnosis of a depressive disorder [62]. Using a Cox proportional hazard regression analysis, this study reported the risk for depression was significantly higher for ADT compared to no treatment with ADT [adjusted HR = 1.93; p = 0.041]. Other small, cross-sectional prostate cancer studies, however, have found no statistical difference in self-reported depressive symptomatology between ADT-treated men compared to men not receiving ADT [50, 57, 63, 64]. The inconsistent findings on the effect of ADT on mood  may have resulted from the cross-section design, insufficient statistical control of variables and biases, lack of statistical power, and other methodological limitations.

Recently, however, three studies with large sample sizes and statistical control of variables have shown a strong association of ADT with a depression diagnosis. A retrospective, observational cohort study (N = 79,930) using the electronic medical record of the Department of Veterans Affairs Healthcare System found that ADT significantly increases the risk for developing a depressive episode over a ten-year period [SHR = 1.50; p < 0.001] using a multivariate competing risks regression model [65]. Using an adjusted Cox proportional hazards analysis and propensity matching and controlling for a past diagnosis of depression, a research group at the Harvard Medical School detected increased risks of new onset depression [AHR = 1.23; p < 0.001] and psychiatric hospitalization [AHR = 1.29; p < 0.001] from androgen deprivation therapy for 6 to 36 months compared to no ADT treatment in men with prostate cancer older than 65 [N = 78,552] from the National Cancer Institute’s Surveillance, Epidemiology, and End Results (SEER)-Medicare-linked database [66]. This study uniquely investigated the time-dependence for adverse effects of ADT on mood demonstrating a dose–response relationship of ADT duration and depression. They also found progressive increases in the cumulative incidence of depression [AHR = 1.37; p < 0.001] and risk of inpatient psychiatric treatment [AHR = 1.47; p < 0.001] with prolonged ADT treatment for 1.0 to 2.5 years. This finding provided evidence for the heightened risk of developing a depressive episode with prolonged ADT treatment [66].

An earlier population-based analysis of the SEER-Medicare database also reported a significantly increased incidence of a depressive disorders in men with prostate cancer after ADT compared to men with prostate cancer not receiving ADT and men without cancer [67]. The observed depressogenic effect of ADT was reduced, however, after adjustment of the Cox proportional hazards regression for a diagnosis of a depressive disorder 12 months before the prostate cancer diagnosis or study entry in addition to other variables including age, ancestry, tumor grade/staging, medical comorbidity, and treatment (radical prostatectomy or radiation therapy) [67]. The findings of these two studies indicate that men with prostate cancer and a history of depression are especially vulnerable to the depressogenic effect of ADT. In 2021, the role of ADT in depression was assessed  in a new study of younger men (aged 40–64 years) with nonmetastatic prostate cancer with and without ADT using the TRICARE insurance data and controlling for a past diagnosis of depression [66, 68]. Kaplan–Meier analyses detected that an increasing risk of new onset depression from ADT over a six-year period, while a Cox proportional hazards regression analysis found that ADT was associated with an increased risk of new-onset depression [AHR = 2.07; p < 0.001] [68]. Again, there was a dose–response positive relationship between the duration of ADT treatment and the risk for depression [68].

### Meta-analyses of androgen deprivation therapy and depression

There have been two meta-analyses strongly supporting the relationship of androgen deprivation therapy with depression. In 2017, a meta-analysis that identified 18 independent studies with a total of 168,756 men with prostate cancer confirmed that ADT significantly increases the risk of depression by 41% [RR = 1.41; p < 0.001] using a random effects model [57]. The significant association of ADT with depression held when the meta-analysis was restricted to studies of localized prostate cancer or a clinical diagnosis of a depressive disorder rather than a depressive inventory by a physician or patient self-report. Continuous ADT did not confer an increased depression risk compared to intermittent ADT [57]. In 2020, another meta-analysis across six studies also confirmed that ADT significantly increases the risk of depression [HR = 1.51, p < 0.0002] [51].

### Androgen receptor antagonist, androgen synthesis inhibitor, and depression

A retrospective study using a large male cohort with prostate cancer (N = 30,069) from the NCI’s SEER-Medicare-linked database and the Texas Cancer Registry (TCR)-Medicare-linked database compared the cumulative incidence of depression, defined by ICD-9/10 criteria, in men who were treated with second generation anti-androgen treatment, which included the CYP17 inhibitor abiraterone and an androgen receptor antagonist (bicalutamide, nilutamide, flutamide, enzalutamide, apalutamide, darolutamide) to men treated with only ADT [69]. Using a multivariate Cox proportional hazards analysis and propensity-scored weighting, the risk of incident depression over a two-year period was substantially higher in the second-generation anti-androgen treatment group compared to the ADT group [HR = 2.26; p < 0.001] and the control group without any treatment [HR = 2.15; p < 0.001]. In men with metastatic prostate cancer, second-generation anti-androgen treatment resulted in the highest rate of incident depression [69]. This important new finding indicates inhibiting androgen receptor signaling in brain regions regulating mood generates a stronger depressogenic action than inducing very low testosterone levels with ADT in men with prostate cancer.

## Testosterone replacement therapy and depression

### Testosterone trials

The Testosterone Trials consisting of seven double-blind, placebo-controlled trials has been the largest investigation to date of the efficacy and benefits of testosterone replacement therapy (TRT) in men older than 65 years who have developed age-related hypogonadism based on strict clinical criteria [15, 70–72]. In the Testosterone Trials cohort of hypogonadal men were characterized  as having two morning total testosterone levels less than 275 ng/dl (9.53 nmol/L SI units), sexual dysfunction, and diminished physical functioning including low vitality.  TRT was confirmed to have the following beneficial effects: (1) libido and sexual activity increased with a lesser improvement in erectile function; (2) hemoglobin levels increased by ~ 1.0 g/L in men with iron-deficiency and chronic anemias; (3) volumetric bone mineral density increased especially in the trabecular bone architecture of lumbar spine vertebrae. It is important to note that the Testosterone Trials found that TRT improved mood and decreased depressive symptoms in hypogonadal men. TRT, however, failed to improve cognitive function and increased coronary artery noncalcified plague volume in coronary arteries by 40 mm^3^/year [15, 70, 71, 73, 74]. This latter finding was not associated with a greater prevalence of cardiovascular events.

### Testosterone treatment and depression

The mood effect of testosterone treatment has been extensively investigated and meta-analyzed in eugonadal and hypogonadal men with depressive symptoms or major depressive disorder with inconclusive results [20, 26, 35, 50, 75–79]. Three early interventional studies of TRT using testosterone gel or intramuscular testosterone undecanoate in men with hypogonadism based on mean total testosterone levels ranging from 230 to 300 ng/dl (7.97–10.40 SI units) reported a significant reduction in depressive symptoms [80–82]. In 2014, a meta-analysis of six studies of testosterone treatment in eugonadal and hypogonadal men, including the above three studies, concluded that TRT improved mood and decreased depressive symptoms in men with low to hypogonadal levels of total testosterone [26].

Randomized, placebo-controlled clinical trials have evaluated the benefit of testosterone treatment in men with major depressive disorder. In 2003, a small RCT study reported that the mean Hamilton score (21.8) in younger men (mean age 46.9 years) with hypogonadism and major depressive disorder refractory to antidepressant medications decreased by ~ 60% when their total testosterone levels were increased from 293 to 789 ng/dl (10.16–27.36 nmol/L SI units) by TRT compared to placebo treatment [83]. Findings from subsequent clinical trials and meta-analyses, however, have  reported inconsistent findings with some studies showing an antidepressant effect of TRT and other studies finding no benefit when men with major depressive disorder were treated with testosterone, although the effect of hypogonadal testosterone levels has not always been analyzed [20, 26, 35, 50, 75–79]. Nevertheless, the largest random effects meta-analysis of testosterone treatment in eugonadal or hypogonadal men with depression included 27 randomized controlled trials and found a significant antidepressant effect of TRT compared to placebo [OR = 2.30; p = 0.004] [79]. In addition, a dose–response relationship was observed with the strongest antidepressant effect occurring when men were treated with testosterone doses higher than 500 mg/week [79].

Interestingly, in two randomized, double-blind, placebo-controlled clinical trials completed in 2009, testosterone treatment of men with dysthymic disorder, which is a milder, but persistent depressive disorder characterized by an early, insidious onset and a chronic course, had a stronger antidepressant effect [84, 85]. In the Vitality Trial of the Testosterone Trials, mild depressive symptoms in hypogonadal men measured by the Patient Health Questionnaire PHQ-9 were significantly reduced by 29% (p = 0.004) by TRT compared to 18% decrease by placebo over a nine-month treatment period [86]. Furthermore, meta-analyses have shown that TRT has a more consistent antidepressant effect in men with less severe, subclinical depression [20, 75, 78, 79, 87]. The TRAVERSE trial is now being completed to determine whether testosterone replacement therapy provides significant benefit in clinical disorders including depression. At present, however, the Testosterone Trials and other studies have only found that TRT can be beneficial in men with dysthymic disorder or subsyndrome depression that does not meet criteria for major depressive syndrome. These findings suggest that hypogonadal levels of testosterone dysregulate mood and induce depressive symptoms that can be ameliorated by testosterone treatment, but TRT is unlikely to be an antidepressant treatment for major depressive disorder.

## Androgen receptor regulation and depression

### Molecular biology of androgen receptor structure

These ubiquitous actions of testosterone and dihydrotestosterone (DHT), the most potent androgen, are signaled by the androgen receptor, AR, (NC-IUPHAR nomenclature: *NR3C4*), which is a member of the superfamily of nuclear steroid hormone receptors and encoded by the AR gene on the long arm of the X chromosome at Xq11-12. The androgen receptor protein consists of a transcriptional regulation domain at the N-terminus that activates or represses target genes, the highly conserved DNA binding domain with two zinc fingers that bind promoter or enhancer DNA consensus sequences of target genes, a small hinge region, and a ligand binding domain at the C-terminus [88, 89]. Testosterone and DHT binding to the ligand binding domain stimulates the androgen receptor protein to assume an active conformation. Testosterone binds to the androgen receptor with a low nanomolar affinity, while the stronger biological action of DHT is mediated by its two-fold higher affinity and five-fold lower rate of dissociation from the AR compared to testosterone. Androgen receptor signaling exerts important biological actions in the testis, prostate, bone, skeletal muscle, heart, vascular smooth muscle, kidney, pulmonary epithelial cells, bone, adipose tissue, and the central nervous system [89, 90]. In the central nervous system, androgen receptors are highly expressed in the arcuate nucleus and other medial basal region of the hypothalamus, the bed nucleus of the stria terminalis and amygdala in limbic pathway, the hippocampus, and the temporal lobe, which are brain regions regulating mood and cognitive function [91, 92]. Androgen receptor expression has been found to be decreased by 2.7-fold in hypothalamus of men with major depressive disorder compared to male controls [93].

### Canonical and non-canonical androgen receptor signaling

Prior to ligand activation, the androgen receptor is sequestered in the cytoplasm where AR is stabilized by heat shock proteins and associated with cytoskeletal proteins and other chaperones [88, 89, 94]. After binding testosterone or DHT, the cytosolic androgen receptor assumes an active confirmation, dissociates from these cytoplasmic proteins, and translocates to the nucleus where the activated AR dimerizes and functions as a ligand-dependent nuclear transcriptional regulator (Fig. 1). The AR then binds to androgen response elements on androgen target genes to activate or repress their expression [88–90]. AR transcriptional regulation is modulated by co-regulators that bind to activated androgen receptors in a ligand-dependent manner to co-activate or co-repress target genes. AR regulation of gene transcription also involves recruitment of transcriptional factors, remodeling of chromatin, and modification of histones.

In addition to the slower genomic actions of the cytosolic AR after translocating to the nucleus, androgen receptors expressed on the cell surface have rapid, non-genomic actions by signaling via downstream calcium, Akt, MAPK-ERK kinase, and protein kinase pathways (Fig. 1), which can regulate synaptic plasticity and have other brain actions [88, 94, 95]. The non-canonical actions of membrane androgen receptors may be coordinated with the canonical actions of androgen receptors in the nucleus. Membrane androgen receptor signaling via non-canonical cascades may be especially important in brain neurons and relevant to antidepressant actions of testosterone by promote cell survival, neurogenesis, synaptic density, and synaptic remodeling in the hippocampus, prefrontal cortex, and other brain regions [96].

### Androgen receptor genetics and depression

Missense mutations in the AR ligand binding result in complete or partial androgen insensitivity syndrome, although mutations in the N-terminal domain encoded by exon 1 have recently been shown to induce resistance to androgen actions [97]. The androgen insensitivity syndrome is the most common genetically driven sex developmental abnormality characterized by a female phenotype in a genetically male 46, XY individual and has reported to increase the risk for depression and be associated with a 36% incidence of depression [98].

Androgen receptor affinity and expression can also be genetically regulated by trinucleotide CAG repeat sequences in exon 1 that vary in length from 9 to 36 repeats [99, 100]. Shorter CAG repeat lengths confer higher affinity and sensitivity of the androgen receptor to testosterone and DHT while longer CAG repeat lengths render the androgen receptor less sensitive to androgens [99, 100]. In men, CAG repeat length is normally distributed with an average of 22 repeats and has been shown to be identical in peripheral leukocytes and brain regions regulating mood and cognitive function [101]. Variation in the AR gene has been associated with male reproductive function, cardiovascular health, prostate cancer, bone density, muscle mass, level of testosterone, and rate of change in testosterone with increasing age [99, 100, 102].

An androgen receptor with higher affinity and sensitivity for testosterone due to shorter CAG repeat length in the presence of low testosterone levels has been associated with depression in men with European or African ancestry [103–105]. However, a study using a logistic regression analysis with stratification for AR CAG repeat length found that the risk for depression was significantly lower in men with a highly sensitive androgen receptor due to short CAG repeats if their testosterone levels were high [103]. This latter finding suggests that men with an androgen receptor having higher sensitivity and transcription activity due to shorter CAG repeats is more strongly impacted by higher testosterone levels and will be more responsive to testosterone replacement therapy. The androgen receptor  may not have important roles in the susceptibility to depression or the positive response to TRT if the androgen receptor has less sensitivity to testosterone due to longer CAG repeats [104, 106–108].

## Neuronal and molecular mechanisms mediating testosterone and depression

Functional neuroimaging studies (fMRI and PET) have found that testosterone can regulate cerebral blood flow and neuronal activity in the amygdala, hippocampus, and frontal and temporal cortex [109–111]. Testosterone can also promote synaptic plasticity and synaptic remodeling in limbic brain neurons expressing the androgen receptor and regulating mood [112–114]. Testosterone activated androgen receptor signaling in the hippocampus has been shown to upregulate neurogenesis, which may promote antidepressant responses in depression [115].

In preclinical research, androgen receptor signaling in brain regions regulating mood has been reported to have anti-stress and antidepressant effects [12]. Orchiectomy abolishes this antidepressant action of testosterone-activated brain AR signaling, while a transgenic mouse with a deletion of the androgen receptor gene has been  shown to develop depressive-like behavior in response to chronic stress compared to wild-type controls [12, 116].  Other research has found that testosterone promotes an antidepressant response by activating androgen receptor signaling via the MAPK-ERK2 cascade in the hippocampus [12, 117].

Deficient serotonergic neurotransmission and reduced serotonin 5-HT1A and 5-HT1B receptor signaling has an important role in the pathophysiology of major depressive disorder and form the basis of the serotonin hypothesis of depression [118]. Increasing synaptic levels of serotonin with selective serotonin reuptake in inhibitors contributes to antidepressant responses in depression [118]. Testosterone treatment upregulates serotonin transporter expression and increases the firing rate of serotonergic dorsal raphe neurons [119, 120] which has been proposed to promote an antidepressant action. Using PET imaging, a recent study has reported that testosterone regulates hippocampal serotonin 5-HT4 receptors and increases brain serotonergic  function [121]. Testosterone can also regulate monoamine oxidase and catechol-o-methyl transferase in amygdala, hippocampus, and other limbic brain areas involved in depression and mediating antidepressant responses [12, 122, 123].

## Summary, conclusions, and future directions

Current findings indicate that low circulating levels of total testosterone meeting stringent clinical criteria for hypogonadism and testosterone deficiency induced by androgen deprivation therapy are associated with increased risk for depression and current depressive symptoms. Furthermore, the Testosterone Trials and other studies have reported that testosterone replacement therapy  may only be beneficial in men with dysthymic disorder or subsyndromal depression that does not meet criteria for major depressive syndrome. These findings suggest that hypogonadal levels of testosterone can dysregulate mood and induce depressive symptoms. The studies reviewed here also suggest that a substantial deficiency in testosterone can cause a depressive-like state that can  respond to TRT. At present, there is no clinical justification to use TRT as an antidepressant treatment for major depressive disorder. Therefore, the benefits of testosterone replacement therapy on major depressive disorder in men with clinically defined hypogonadism remains uncertain and will hopefully be elucidated by the TRAVERSE Trial and other ongoing research.

**Fig. 2:**
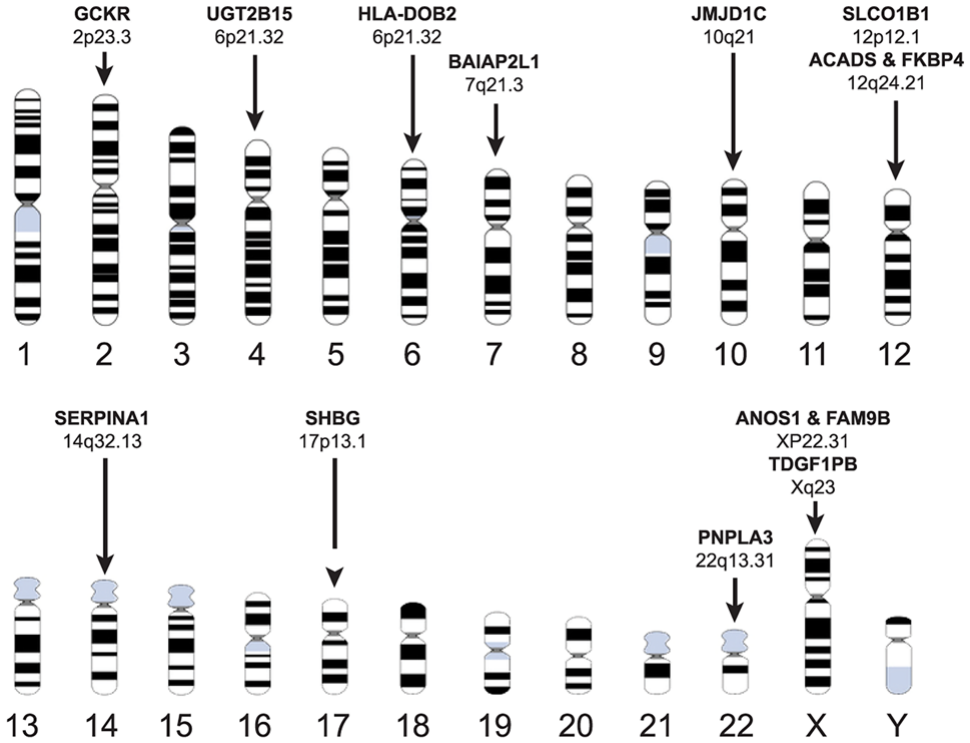
Chromosome idiogram map of gene variants that have significant genome-wide association with testosterone. The localization of testosterone gene variants to specific chromosomes is depicted. Gene variants were identified to have genome-wide significance for regulating testosterone based on GWAS studies of morning total testosterone levels in the UK Biobank and Million Veteran Program [21–23, 127]

Important considerations are that major depressive disorder is a clinically heterogeneous phenotype with depressed individuals differing in inherited polygenic determinants, onset and clinical course, symptom complexes, and comorbidities that contribute to potential multifactorial differences in pathophysiology. Furthermore, polygenic mechanisms are likely to be critical to the biological heterogeneity that influences testosterone-depression interactions. A recent study has identified certain regulatory variants linked to genetic risk for major depressive disorder in a GWAS, which include hippocampal transcription factors enriched for ZMIZ1, a zinc finger co-activator that increases ligand-dependent transcription of the androgen receptor and promotes androgen receptor sumoylation required for androgen receptor function [124]. Research on male twins has provided heritability estimates of 57–58% for total testosterone [125, 126]. Genome-wide association studies (GWAS) from the UK Biobank and other large cohorts have identified the SNP-based heritability for total testosterone to be ~ 20% and free testosterone to be ~ 15% [21–23, 127]. Recent GWAS research has identified significant associations of GCKR, BAIAP2L1, JMJD1C, FKBP4, SERPINA1, SHBG, FAM9B, and other gene variants with total testosterone levels [21–23, 127] (Fig. 2). Polygenic scores derived from testosterone GWAS data predict testosterone levels and their association with important phenotypes and clinical disorders. As mentioned earlier, a recent investigation of 169,886 male participants (40–69 years) without a history of depression in the prospective UK Biobank study reported that hypogonadal men with very low total testosterone levels (< 6.0 nmol/L; 173 ng/dl) had high incidence of developing a major depressive episode over a five-year period [adjusted OR = 1.60] [33]. The association of major depressive disorder incidence with testosterone levels in the severe hypogonadal range had the largest effect size among the 57 laboratory tests analyzed in the UK Biobank. Using the UK Biobank genetic database, Mendelian randomization analyses found a beneficial, protective effect of genetically predicted, lifelong free testosterone on depression in men [22]. A genetically informed precision medicine approach using genes regulating testosterone levels and androgen receptor sensitivity will likely provide critical insight into the role of testosterone in depression.

## Abbreviations

ACADS: Acyl-CoA dehydrogenase short chain
ACTH: Adrenocorticotropin stimulating hormone
ANOS1: Anosmin 1
5-Adiol: Androstenediol
Akt: Ak strain transforming protein/Protein kinase B
ADT: Androgen deprivation therapy
AR: Androgen receptor
BAIAP2L1: BAR/IMD domain containing adaptor protein 2 like 1 gene
BDI: Beck Depression Inventory
cyclic AMP: Cyclic adenosine monophosphate
CYP17: Cytochrome P450 17α-hydroxylase/17,20-lyase
CAG repeats: Cytosine-adenine-guanine repeat sequences
DHEA: Dehydroepiandrosterone
DHEA-S: Dehydroepiandrosterone-sulfate
DHT: Dihydrotestosterone
FAM9B: Family with sequence similarity 9 member B
FKBP4: FK506-binding protein 4 gene
fMRI: Functional magnetic resonance imaging
HIMS: Health in Men Study
GWAS: Genome-wide association studies
GCKR: Glucokinase regulator
GnRH: Gonadotropin-releasing hormone
HAM-D: Hamilton depression score
HLA-DOB2: HLA class II histocompatibility complex, class II, DO beta
HPG axis: Hypothalamic-pituitary–gonadal axis
ICD-9: International Classification of Diseases, Ninth Revision
ICD-10: International Classification of Diseases, Tenth Revision
JMJD1C: Jumonji domain containing 1C gene
KISS1R: Kisspeptin receptor 1
LC–MS/MS: Liquid chromatography with tandem mass spectrometry
LH: Luteinizing hormone
MVP: Million Veteran Program
MAPK-ERK: Mitogen-activated protein kinase/Extracellular -signal-regulated kinase
SEER: National Cancer Institute’s Surveillance, Epidemiology, and End Results
NC-IUPHAR: Nomenclature and Standards Committee of the International Union of Basic and Clinical Pharmacology
PET: Positron emission tomography
PNPLA3: Patatin like phospholipase domain containing 3
5-HT1A: Serotonin 5-hydroxytryptamine receptor 1A
5-HT1B: Serotonin 5-hydroxytryptamine receptor 1B
SERPINA1: Serpin family A member 1 gene
SHBG: Sex hormone binding globulin
SLCOB1: Solute carrier organic anion transporter family member 1B1
TDGF1PB: Teratocarcinoma-derived growth factor 1 pseudogene
TRT: Testosterone replacement therapy
TRAVERSE: Testosterone Replacement Therapy for Assessment of Long-term Vascular Events
UGT2B15: UDP glucuronosyltransferase family 2 member B15
UK Biobank: United Kingdom Biobank
